# Supplementary material for: MetaRibo-Seq measures translation in microbiomes
Source: Nat Commun. 2020 Jun 29;11:3268. doi: 10.1038/s41467-020-17081-z (PMC7324362; doi:10.1038/s41467-020-17081-z)
Supplement: Supplementary file 10 — Supplementary Data 7 [file 41467_2020_17081_MOESM10_ESM.zip › File2/Confidence_VeryHigh_Taxonomy/368502_out.krona.html]

Javascript must be enabled to view this page.

members
magnitude
magnitudeUnassigned
count
unassigned
taxon
rank

368502\_out

125


SRS098571\_contig\_number\_contig-100\_19204.19204
1

2
124
superkingdom

phylum
124
1239

species

SRS011084\_contig\_number\_30690SRS013521\_contig\_number\_contig-100\_8013.68802SRS015579\_contig\_number\_3047SRS049995\_contig\_number\_4508SRS050801\_contig\_number\_contig-100\_1120.1121SRS053356\_contig\_number\_18642SRS054905\_contig\_number\_21352SRS056273\_contig\_number\_3147SRS063190\_contig\_number\_13321SRS075984\_contig\_number\_18376SRS077392\_contig\_number\_contig-100\_19949.176924SRS078176\_contig\_number\_contig-100\_5460.84992SRS1041039\_contig\_number\_25316SRS1041137\_contig\_number\_28129SRS1041141\_contig\_number\_12109SRS1041145\_contig\_number\_6368SRS142542\_contig\_number\_10984SRS147557\_contig\_number\_11992SRS148196\_contig\_number\_contig-100\_8525.184589SRS148817\_contig\_number\_contig-100\_463.94361SRS150029\_contig\_number\_contig-100\_3866.113836SRS893373\_contig\_number\_18802
22
1263003


SRS020869\_contig\_number\_12392
1
species
1263000

class
101
186801

order
101
186802

family

SRS013476\_contig\_number\_contig-100\_17511.150123
6
216572
1

459786
genus
5

1235797

SRS013687\_contig\_number\_23398
1
species

1263547
species

SRS015782\_contig\_number\_contig-100\_28680.28681SRS103987\_contig\_number\_contig-100\_30874.72574SRS143876\_contig\_number\_contig-100\_31662.31662
3

1945593

SRS143895\_contig\_number\_29012
1
species

2
family
541000

genus
1
216851


SRS054352\_contig\_number\_9643
1
species
853

1898205

SRS050752\_contig\_number\_1409
1
species

31979
family

SRS048060\_contig\_number\_contig-100\_764.765
1

species

SRS011134\_contig\_number\_contig-100\_5263.29800SRS011239\_contig\_number\_contig-100\_1744.159099SRS011302\_contig\_number\_13296SRS012273\_contig\_number\_41377SRS012969\_contig\_number\_412SRS013098\_contig\_number\_17562SRS013098\_contig\_number\_21330SRS013965\_contig\_number\_29845SRS014979\_contig\_number\_contig-100\_16423.110554SRS015431\_contig\_number\_74384SRS015663\_contig\_number\_contig-100\_9841.130989SRS015694\_contig\_number\_8789SRS016018\_contig\_number\_7125SRS016335\_contig\_number\_42443SRS018351\_contig\_number\_15657SRS018427\_contig\_number\_13759SRS018836\_contig\_number\_11878SRS019068\_contig\_number\_72771SRS019445\_contig\_number\_26450SRS019685\_contig\_number\_37158SRS021484\_contig\_number\_20823SRS021948\_contig\_number\_29063SRS022071\_contig\_number\_35635SRS022524\_contig\_number\_contig-100\_15787.47494SRS023526\_contig\_number\_12266SRS023914\_contig\_number\_25690SRS024132\_contig\_number\_contig-100\_13432.218008SRS024435\_contig\_number\_26811SRS042966\_contig\_number\_contig-100\_18291.98965SRS043701\_contig\_number\_contig-100\_16770.60241SRS045004\_contig\_number\_contig-100\_18459.112477SRS045645\_contig\_number\_30771SRS045826\_contig\_number\_14698SRS048870\_contig\_number\_18552SRS049896\_contig\_number\_contig-100\_5617.37791SRS049959\_contig\_number\_18500SRS050925\_contig\_number\_contig-100\_15259.95750SRS052697\_contig\_number\_contig-100\_7743.204123SRS053398\_contig\_number\_contig-100\_16748.96580SRS055017\_contig\_number\_17539SRS058070\_contig\_number\_13352SRS062654\_contig\_number\_contig-100\_28048.70813SRS063040\_contig\_number\_contig-100\_11766.170335SRS063518\_contig\_number\_8070SRS064276\_contig\_number\_20031SRS064757\_contig\_number\_13541SRS065504\_contig\_number\_contig-100\_5115.198401SRS074586\_contig\_number\_2145SRS075078\_contig\_number\_4300SRS075341\_contig\_number\_20544SRS075773\_contig\_number\_36558SRS075821\_contig\_number\_contig-100\_25885.76103SRS077024\_contig\_number\_14064SRS077194\_contig\_number\_contig-100\_13317.94223SRS077641\_contig\_number\_contig-100\_5966.47306SRS077849\_contig\_number\_9958SRS078242\_contig\_number\_23506SRS078419\_contig\_number\_26799SRS098571\_contig\_number\_7471SRS098644\_contig\_number\_37972SRS100907\_contig\_number\_contig-100\_13437.13438SRS101376\_contig\_number\_6374SRS1041031\_contig\_number\_23133SRS1041032\_contig\_number\_11487SRS1041092\_contig\_number\_15872SRS1041133\_contig\_number\_contig-100\_1053.144691SRS1041139\_contig\_number\_3239SRS1041140\_contig\_number\_17365SRS1041157\_contig\_number\_4145SRS104400\_contig\_number\_44291SRS104912\_contig\_number\_10619SRS142503\_contig\_number\_45611SRS143417\_contig\_number\_contig-100\_2690.190208SRS143466\_contig\_number\_661SRS143523\_contig\_number\_contig-100\_4510.125277SRS144183\_contig\_number\_32757SRS144506\_contig\_number\_contig-100\_13573.120878SRS144537\_contig\_number\_40786SRS146812\_contig\_number\_contig-100\_21265.168046SRS146813\_contig\_number\_contig-100\_4185.53227SRS147346\_contig\_number\_44296SRS147614\_contig\_number\_8072SRS148253\_contig\_number\_4212SRS148424\_contig\_number\_27408SRS892882\_contig\_number\_8818SRS893172\_contig\_number\_12649SRS893187\_contig\_number\_contig-100\_5209.76234SRS893358\_contig\_number\_contig-100\_5097.42824SRS893366\_contig\_number\_15707SRS893383\_contig\_number\_36148SRS971275\_contig\_number\_4915SRS971276\_contig\_number\_27835
92
172733
